# Functional Isolation of Tumor-Initiating Cells using Microfluidic-Based Migration Identifies Phosphatidylserine Decarboxylase as a Key Regulator

**DOI:** 10.1038/s41598-017-18610-5

**Published:** 2018-01-10

**Authors:** Yu-Chih Chen, Brock Humphries, Riley Brien, Anne E. Gibbons, Yu-Ting Chen, Tonela Qyli, Henry R. Haley, Matthew E. Pirone, Benjamin Chiang, Annie Xiao, Yu-Heng Cheng, Yi Luan, Zhixiong Zhang, Jason Cong, Kathryn E. Luker, Gary D. Luker, Euisik Yoon

**Affiliations:** 1 Department of Electrical Engineering and Computer Science, University of Michigan, 1301 Beal Avenue, Ann Arbor, MI 48109-2122 USA; 2 Comprehensive Cancer Center, University of Michigan, 1500 E. Medical Center Drive, Ann Arbor, MI 48109 USA; 3 Forbes Institute for Cancer Discovery, University of Michigan, 2800 Plymouth Rd., Ann Arbor, MI 48109 USA; 4 Center for Molecular Imaging, Department of Radiology, University of Michigan, 109 Zina Pitcher Place, Ann Arbor, MI 48109-2200 USA; 5 Computer Science Department UCLA, Boelter Hall, Los Angeles, CA 90095-1596 USA; 6 Department of Microbiology and Immunology, University of Michigan, 109 Zina Pitcher Place, Ann Arbor, MI 48109-2200 USA; 7 Department of Biomedical Engineering, University of Michigan, 2200 Bonisteel, Blvd., Ann Arbor, MI 48109-2099 USA

## Abstract

Isolation of tumor-initiating cells currently relies on markers that do not reflect essential biologic functions of these cells. We proposed to overcome this limitation by isolating tumor-initiating cells based on enhanced migration, a function tightly linked to tumor-initiating potential through epithelial-to-mesenchymal transition (EMT). We developed a high-throughput microfluidic migration platform with automated cell tracking software and facile recovery of cells for downstream functional and genetic analyses. Using this device, we isolated a small subpopulation of migratory cells with significantly greater tumor formation and metastasis in mouse models. Whole transcriptome sequencing of migratory versus non-migratory cells from two metastatic breast cancer cell lines revealed a unique set of genes as key regulators of tumor-initiating cells. We focused on phosphatidylserine decarboxylase (PISD), a gene downregulated by 8-fold in migratory cells. Breast cancer cells overexpressing PISD exhibited reduced tumor-initiating potential in a high-throughput microfluidic mammosphere device and mouse xenograft model. PISD regulated multiple aspects of mitochondria, highlighting mitochondrial functions as therapeutic targets against cancer stem cells. This research establishes not only a novel microfluidic technology for functional isolation of tumor-initiating cells regardless of cancer type, but also a new approach to identify essential regulators of these cells as targets for drug development.

## Introduction

Studies in breast cancer and other malignancies demonstrate that tumor initiation, progression, and metastasis are driven by tumor-initiating cells (TICs), also known as cancer stem cells. TICs constitute a subset of malignant cells capable of unlimited self-renewal and differentiation into cancer cells that form the bulk of a tumor^1–3^. Based on data from animal models and patients with multiple types of malignancies, a central mechanism to generate TICs is epithelial-to-mesenchymal transition (EMT)^4–7^. EMT encompasses numerous steps through which polar epithelial cells lose epithelial characteristics and gain properties of mesenchymal cells, such as increased migration and invasion. The fundamental link between TICs and EMT strongly suggests enhanced migration as a hallmark function of TICs that can be used to identify these cells.

Analyzing TICs remains challenging due to relative rarity of these cells in most cancers and the complexity of identifying them amongst heterogeneous populations of malignant cells in a tumor. Currently, investigators most commonly identify breast cancer TICs by cell surface (CD24^−/low^/CD44^+^) or enzymatic markers (aldehyde dehydrogenase, ALDH^br^)^8,9^. However, marker-based approaches for TICs suffer from several limitations: i) a modest enrichment for TICs with a large portion of recovered cells lacking the ability to form new tumors^10^; ii) inconsistency across different cancer types and even within the same type of cancer^9–12^; and iii) limited relation to actual functions of TICs or patient prognosis^13,14^. Since these markers do not test for essential functions of TICs, there is an unmet need to improve techniques to enrich for TICs^13^. Identification of functional markers for TICs will advance our understanding of cancer biology and point to new targets for drug development.

To advance studies of TICs, we developed a high-throughput microfluidic platform to isolate TICs in breast cancer by the EMT property of enhanced cell migration. This approach enriches TICs based on an essential function rather than empirically-defined markers. In this microfluidic device, we place single cancer cells at the entrance of microchannels, enabling us to identify and recover subpopulations with greatest migration towards a chemoattractant (serum). The large number of channels in this microfluidic device allows us to retrieve sufficient numbers of cells for functional and genomic analyses, a key advantage of our system over prior microfluidic migration devices. We identified a small subset of migratory cells from two different triple negative breast cancer cell lines. In mouse models, migratory cells from each cell line formed more tumors and metastasized to a significantly greater extent than matched non-migratory cells, showing that enhanced migration enriches for TICs. Whole transcriptome sequencing (mRNA Next Generation Sequencing) of migratory versus non-migratory cells revealed a unique set of differentially-expressed genes as potential regulators of TICs. Among candidate genes, we validated phosphatidylserine decarboxylase (PISD), a gene highly downregulated in migratory cells, as a novel regulator of TIC cells in breast cancer. Increasing expression of PISD in breast cancer cells not only reduces primary tumor growth but also causes mitochondrial fragmentation, loss of mitochondrial mass, and perturbations in cellular metabolism. For the first time, this research establishes PISD as novel regulator of TICs in breast cancer and highlights mitochondrial functions and dynamics as potential therapeutic targets specifically against TICs. The strong relationship between EMT and TICs across almost all epithelial cancers suggests that our approach may become a general technology to isolate TICs in multiple malignancies beyond breast cancer.

## Results

### Migration-based TIC enrichment

**Figure 1:**
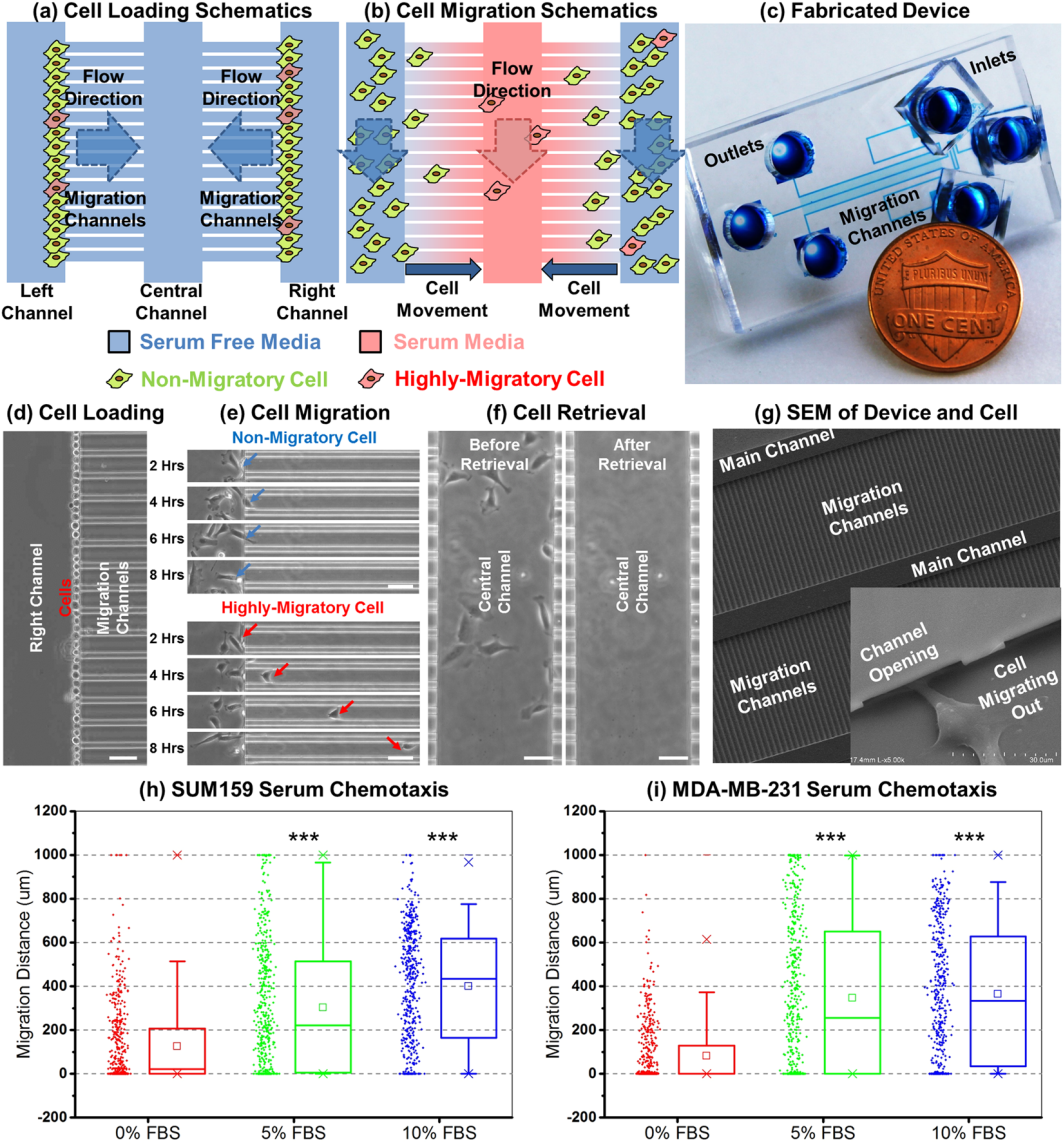
Microfluidic high-throughput (HT) cell migration chip for migration based selection. (**a**,**b**) Schematics of the chip. (a) We initially load cells into the left/right channels. (**b**) After cell loading, serum is introduced as a chemoattractant for cell migration into the central channel. Highly migratory cells move to the central channel from the left/right channels perfused with serum-free media. Finally, we selectively retrieve highly-migratory cells from the central channel and non-migratory cells from the right/left channels, respectively, by trypsinization. (**c**) Photo of a fabricated 900-channel device. Inlets are on the right side, outlets are on the left side, and the migration channels are in the center. The penny is used for scale. (**d**–**f**) SUM159 cell loading, migration, and retrieval on-chip. (**d**) Uniform initial cell loading at the entrance of migration channels. (**e**) 8-hour time-lapse tracking of cell migration from the left loading channel into migration channels. A highly-migratory cell has moved more than 300 µm, while non-migratory cells remain at the loading position. (**f**) Successful cell retrieval by flowing trypsin in the central channel for 5 minutes. (scale bar: 50 µm) (**g**) SEM of the chip with higher magnification of a cell entering a channel. (**h**,**i**) Graphs show positions of individual cells and box plot and whiskers summaries for migration of SUM159 (**h**) and MDA-MB-231 (**i**) cells toward both 5% and 10% fetal bovine serum solution added in the central channel. The elevated migration of chemotaxis validates the migration experiment setup. (n = 900 channels). ***refers to P < 0.001.

To isolate sufficient numbers of migratory breast cancer cells for subsequent analyses, we designed a cell migration platform with left/central/right main channels for cell loading/retrieval and individual channels for selecting migratory cells (Fig. 1(a–c)). Since mammalian cancer cell diameter (10–15 µm) is larger than the migration channel height (5 µm), cells are initially positioned at the entrance of migration channels (Fig. 1(d)). Loaded cells adhere and then migrate toward the central channel in response to a gradient of serum (Fig. 1(e) and Supplementary Video 1)^15^. To assess cell migration in hundreds of channels, we developed automated image analysis software that: (1) imports a TIFF image; (2) segments migration channels from the whole image; (3) identifies cells; (4) excludes false events (debris and noise); and (5) calculates migration distance. We observed marked heterogeneity of migration of SUM159 and MDA-MB-231 human breast cancer cells in the device with subpopulations reaching the central channel (migratory cells) or remaining in the loading channels (non-migratory cells). We also developed the capability to selectively retrieve migratory and non-migratory cell populations by flowing trypsin in the different main chambers after assays, allowing functional assays with recovered cells (Fig. 1(f)). A SEM image of the migration chip and a cell migrating out from migration channel are shown in Fig. 1(g), and results of breast cancer cell lines MDA-MB-231 and SUM159 migrating toward serum are shown in Fig. 1(h,i). Overall, these studies validate the technology used to isolate and recover migratory breast cancer cells as a potential label-free approach to identify TICs.

### Migratory cell population enriches for TICs

**Figure 2:**
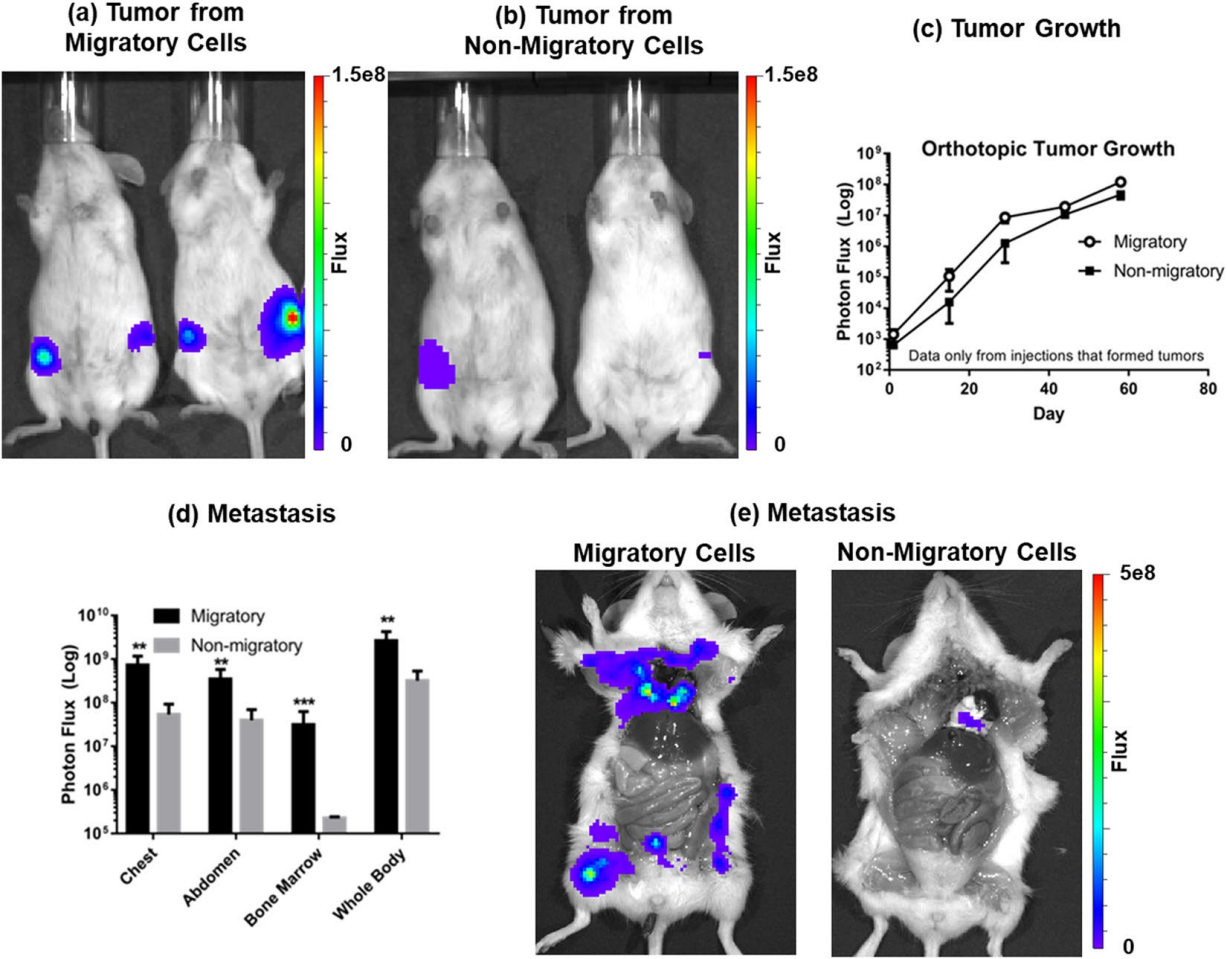
Increased tumor initiation, growth, and metastasis of MDA-MB-231 migratory cells. (**a**,**b**) Migratory MDA-MB-231 breast cancer cells have increased frequency of tumor formation and produce larger tumors. Bioluminescence images of female NSG mice 50 days after orthotopic implantation of 100 migratory or non-migratory cells recovered from the migration device. Scale bar denotes range of photons displayed on pseudocolor scale with red and blue denoting highest and lowest values, respectively. Note that the minimum display is 2 logs lower for mice with non-migratory cells to show any signal. If the minimum is set to same level as tumors from migratory cells, no signal is evident. Conversely, if the minimum is set to the value used for non-migratory cells, light shines over the entire mouse in the migratory group. (**c**) Tumor growth of highly-migratory and non-migratory MDA-MB-231 cells. Graphs show mean and SEM data *only* from mice that formed tumors in each group. Bioluminescence imaging shows greater growth of migratory cancer cells (p < 0.01 by area-under-the-curve for photon flux). (**d**) Metastasis induced by highly-migratory and non-migratory MDA-MB-231 cells. Migratory cancer cells produced greater metastases throughout mice as determined by bioluminescence imaging (**p < 0.01; ***p < 0.005). Note log scale on graphs. (**e**) Representative bioluminescence images of mice injected with 100 MDA-MB-231 migratory or non-migratory cells, depicting greater metastasis for mice injected with the highly migratory cells.

To test the hypothesis that migratory cells recovered from our device are enriched for TICs, we implanted either 100 migratory or non-migratory breast cancer cells (SUM159 or MDA-MB-231) per mouse orthotopically into mammary fat pads of female NSG mice. Approximately two months after implantation, 9/10 injections of migratory MDA-MB-231 cells produced tumors as compared with only 4/10 for non-migratory cells. Calculating the stem cell frequency results in 1 stem cell in every 43 cells for migratory and every 196 cells for non-migratory cells, respectively, representing ~5-fold enrichment for TICs in the migratory population (Table 1). Considering only tumors that formed in each group, tumors from migratory MDA-MB-231 cells grew to a greater extent and generated more metastases than non-migratory cells (Fig. 2(a,e)). We observed even greater enrichment for TICs in the migratory subset of SUM159 cells. After 11 weeks, 9/10 injections of 100 migratory SUM159 cells produced tumors, while 0/10 tumors formed from non-migratory cells (Table 1, Supplemental Fig. S1(a–b)). In addition, only the migratory SUM159 cells metastasized as determined by bioluminescence imaging (Supplemental Fig. S1(c)). These data demonstrate that our microfluidic migration technology enables marker-free enrichment of TICs.

**Table 1:** Frequency of Tumor Formation: Migratory versus Non-Migratory Cells (100 cells per implantation).

**Cell type**	Migratory Cells (Tumors/Implants)	1/stem cell frequency with confidence intervals	Non-Migratory Cells (Tumors/Implants)	1/stem cell frequency with confidence intervals
MDA-MB-231	9/10	43.4 (94.4–19.4)	4/10	196 (527–72.7)
SUM159	9/10	43.4 (94.4–19.4)	0/10	0

### Whole transcriptome sequencing for differentially expressed genes in functionally enriched TICs

**Figure 3:**
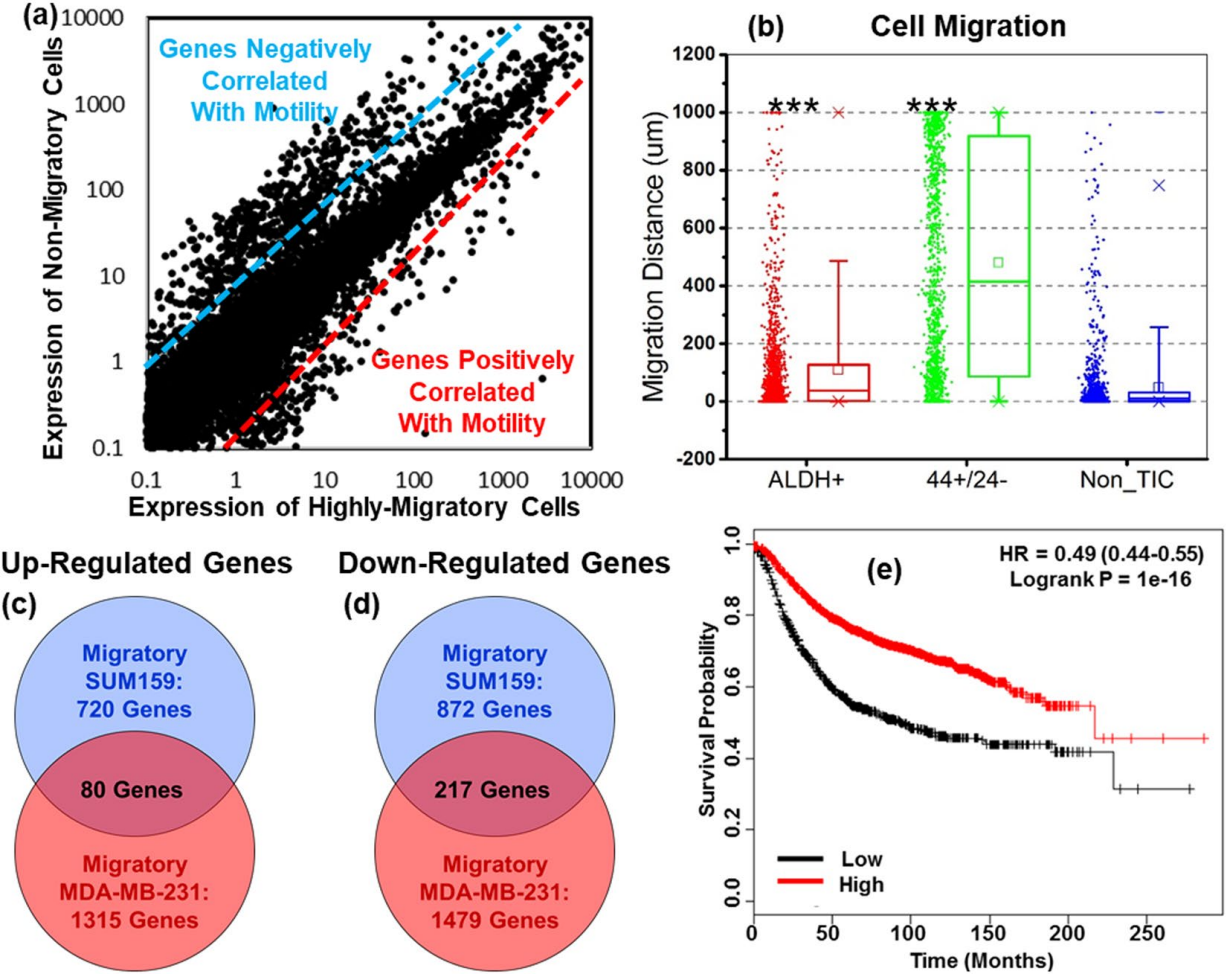
Whole transcriptome sequencing reveals genes differentially expressed between MDA-MB-231 migratory and non-migratory cells. (**a**) Gene expression of migratory and non-migratory MDA-MB-231 cells determined by next generation sequencing. Each dot represents a gene. Genes in the upper left and lower right corners correlate negatively and positively with migratory cells, respectively. (**b**) 5% serum chemo-attraction migration assay of ALDH^br^, CD24^−/low^/CD44^+^ and non-TIC of MDA-MB-231 cells. Both ALDH^br^ and CD24^−/low^/CD44^+^ cells have higher motility than non-TICs. (n = 1,200 channels). ***refers to P < 0.001. (**c**,**d**) Comparison of significantly up-regulated and down-regulated genes of SUM159 and MDA-MB-231 cells. (**e**) The Kaplan-Meier plot shows that low levels of PISD expression correlate with reduced relapse-free survival (RFS) in breast cancer.

To discover differences in gene expression between migratory and non-migratory breast cancer cells, we performed transcriptome analysis for SUM159 and MDA-MB-231 breast cancer cells sorted in the migration device. Among 23,000 genes (Fig. 3(a) and Supplemental Fig. S2(a)), we identified differentially expressed genes distinguishing migratory from non-migratory cells (Table 2and Supplementary Data 1). Using gene set enrichment analysis (GSEA), we found many top-ranked sets have associations with cell migration, chemotaxis, and cell proliferation (Table 3). To determine overlap of TICs selected by migration versus conventional markers (ALDH^br^ and CD24^−/low^/CD44^+^), we compared differentially expressed genes we identified for migratory cells with those reported in literature^16^. Although both ALDH^br^ and CD24^−/low^/CD44^+^ SUM159 cells have higher migration than non-TICs in our assay (Fig. 3(b) and Supplemental Fig. S2(b)), we found few genes overlapping with both ALDH^br^ and CD24^−/low^/CD44^+^ cell populations (Supplemental Fig. S2(c,d), S3(a,b), and Supplementary Data 1).

**Table 2:** The Expression of Top-Ranked Up- and Down-Regulated Genes in Migratory Cells as Compared to Non-Migratory Cells.

**Up-regulated Genes (log2(fold change))**			**Down-regulated Genes (log2(fold change))**		
Genes	SUM159	MDA-MB-231	Genes	SUM159	MDA-MB-231
GHRL	3.45	2.87	PISD	−3.58	−2.83
SERPINE1	4.75	2.35	CXCL8	−2.58	−5.68
PER1	1.91	1.58	CXCL2	−2.51	−2.57
RGS2	2.27	1.53	GDF15	−2.39	−3.49
SNAI2	1.25	2.17	CELSR3	−2.12	−3.45
PMEPA1	2.81	1.19	KYNU	−2.13	−2.11
THBS1	1.50	1.12	CXCL3	−2.10	−2.28
SLC7A2	2.63	1.10	ISG20	−3.13	−1.92
FSTL3	1.43	1.10	ZMIZ1AS1	−1.87	−2.47
HSF2BP	3.13	1.08	PTGS2	−1.84	−4.86
PHLDB1	3.19	1.06	F2RL2	−1.92	−1.77
UBAP2	1.43	1.03	FAIM3	−2.91	−1.64
FKBP5	3.13	1.00	CEMIP	−1.63	−1.76
COL4A1	2.91	1.00	NR4A2	−1.63	−1.65
DMBT1	0.98	4.30	RNF170	−1.60	−6.17
NEIL2	1.14	0.95	MX2	−1.55	−1.86
NFIA	0.92	1.09	CD24	−1.55	−1.57
KRT7	0.90	0.98	CSF1	−1.46	−2.33
COL4A2	2.31	0.86	CA11	−1.60	−1.45
TUBA4A	0.85	1.16	SYT17	−1.67	−1.43

**Table 3:** Relevant GO Biological Processes of highly-migratory cells based on GSEA Figures.

**GO Biological Process**	**DEGenes/Gene Set**	**P-value**
Cell adhesion	208/803	1.00E-24
Response to stimulus	767/5042	1.00E-24
Cell migration	192/187	2.27E-22
Regulation of cell migration	128/453	1.38E-19
Cell motility	194/837	1.06E-19
Regulation of locomotion	137/516	1.38E-18
Regulation of response to stimulus	420/2435	1.22E-18
Cellular response to stimulus	645/4329	3.57E-18
Locomotion	239/1154	4.54E-18
Regulation of cell motility	130/480	4.06E-18
Movement of cell or subcellular component	250/1247	4.46E-17
Response to external stimulus	291/1152	1.95E-17
Positive regulation of locomotion	90/284	1.95E-16
Positive regulation of cell motility	86/273	2.19E-15
Positive regulation of cell migration	85/268	2.19E-15
Cell proliferation	241/1265	2.52E-13
Inflammatory response	96/344	1.70E-13
Response to wounding	149/653	1.46E-13
Regulation of cell proliferation	193/966	5.52E-12
Chemotaxis	121/532	2.76E-10

Our migration-based enriched cells have higher tumor-initiating potential but a different molecular signature than conventionally defined TICs, providing an opportunity to identify novel regulators of TICs and potential therapeutic targets. We first prioritized sequencing data by identifying top-ranked over- and under-expressed genes between migratory and non-migratory cells in two different breast cancer cell lines (SUM159 and MDA-MB-231). Among overlapping genes (significantly over- or under-expressed in migratory cells from both cell lines) (Fig. 3(c,d)), we used publicly available databases to determine correlations with prognosis in breast cancer (i.e. lower expression of a gene in migratory TICs correlates with significantly worse survival in patients)^17^. Combining transcriptome analysis and data mining for patient outcomes, we identified candidate genes either over- or under-expressed in highly migratory cells that were concordant with significant differences in survival of patients with breast cancer. We focused on phosphatidylserine decarboxylase (PISD), a top-ranked gene down-regulated by approximately 8-fold in migratory TICs from both SUM159 and MDA-MB-231 cells (p < 0.00005). PISD, a mitochondrial enzyme that converts phosphatidylserine (PS) to phosphatidylethanolamine (PE), has not previously been associated with TICs. Data from the TCGA for breast cancer accessed through Oncomine showed a trend toward lower expression of PISD in invasive ductal carcinoma relative to normal breast tissue, although extensive overlap exists (Supplementary Fig. S4). Breast cancer patients with low PISD have significantly reduced relapse free survival (RFS) (Fig. 3(e)). Concordance between decreased levels of PISD in migratory cells and poorer survival supports further analysis of PISD as a potential new regulator of TICs in breast cancer.

### Overexpression of PISD reduces *in vitro* TIC phenotypes and tumor growth

**Figure 4:**
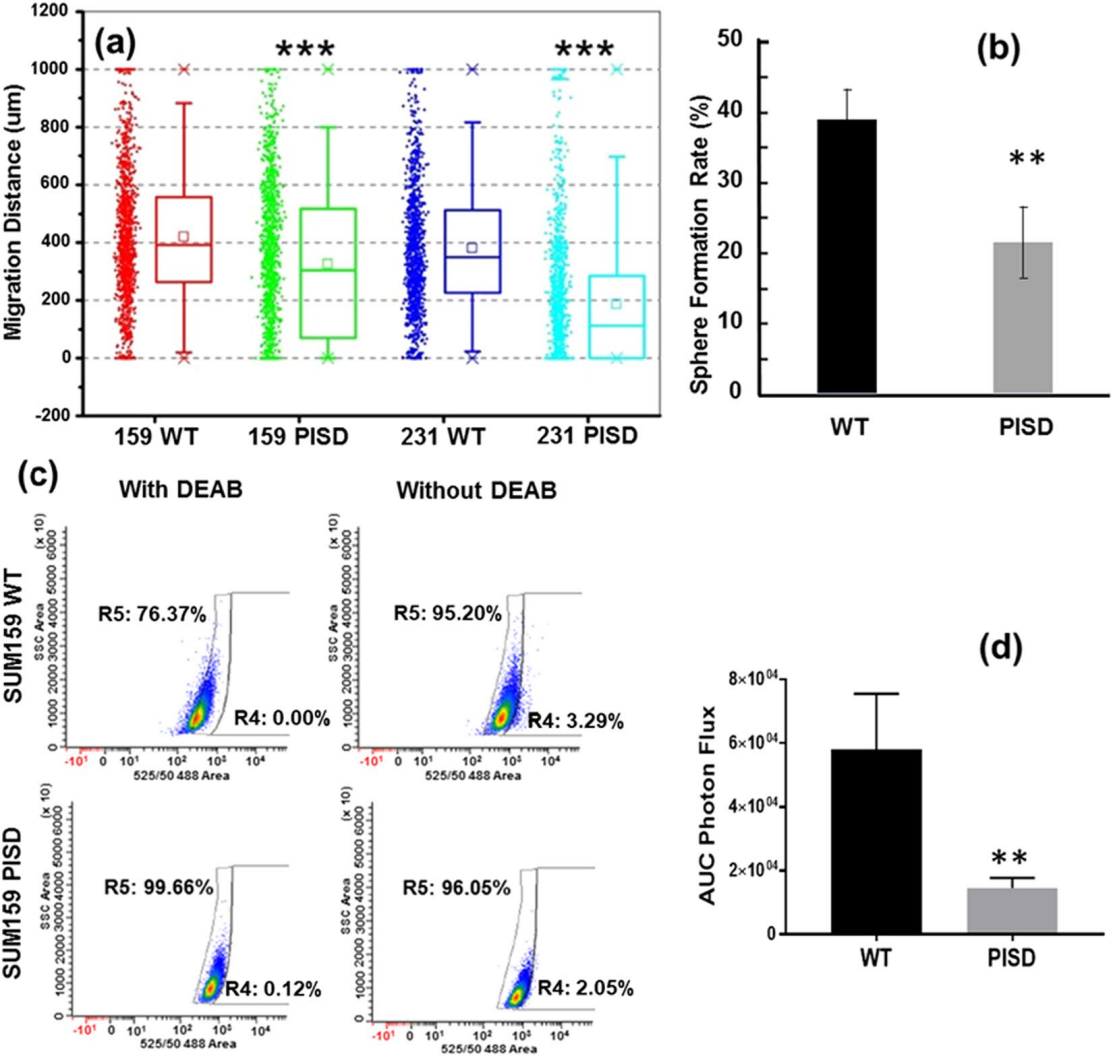
Overexpression of PISD significantly reduces cell migration, tumor sphere formation, ALDH-positive cells, and tumor growth. (**a**) Stable expression of PISD significantly reduces migration of SUM159 and MDA-MB-231 cells toward 5% serum. (n = 1,200 channels). ***refers to P < 0.001. (**b**) SUM159-PISD cells have lower tumor sphere formation rate than wild type SUM159 cells. (n = 3 devices, ~1,000 single cells per device) **refers to P < 0.01. (**c**) PISD expression significantly reduces ALDEFLUOR staining in SUM159 cells. DEAB inhibits ALDH activity, which defines the control gate. (**d**) Area-under-the-curve photon flux for wild type SUM159 and cells stably expressing PISD. Graphs show mean ± SEM (n = 6). **refers to P < 0.01.

We initially tested effects on migration of SUM159 cells after transient transfection of PISD or vector control. Overexpression of PISD reverses reduced expression of this gene in migratory cells. Transient overexpression of PISD significantly decreased migration toward serum in our migration device (Supplemental Fig. S5). Stable expression of PISD fused to fluorescent protein mCherry (PISD-mCherry) also significantly reduced migration of SUM159 and MDA-MB-231 cells relative to wild-type control cells expressing unfused mCherry (Fig. 4(a) and Supplementary Video 2,3). We also measured effects of PISD on formation of tumor-spheres from single cells, an assay used extensively to assess tumor-initiating potential of cancer cells *in vitro* ^9^. Using our previously developed single-cell microfluidic chip, we trapped thousands of single cells in non-adherent wells and enumerated spheres larger than 40 µm after 14 days^18^. SUM159-PISD cells formed significantly fewer spheres than parental SUM159 cells (p < 0.01) (Fig. 4(b)). MDA-MB-231 cells did not form spheres, independent of PISD overexpression.

We also assessed effects of expressing PISD on TICs determined by flow cytometry for established markers ALDH^br^ and CD24^−/low^/CD44^+^. Expression of PISD reduced ALDH^br^ SUM159 cells (Fig. 4(c)). There were insufficient ALDH^br^ MDA-MB-231 cells (<0.5%) to evaluate effects of PISD on this cell line. By comparison, PISD did not alter the high percentage of CD24^−/low^/CD44^+^ cells in either cell line. Discordance between populations defined by ALDH^br^ and CD24^−/low^/CD44^+^ has been reported previously with these markers proposed to define more epithelial and mesenchymal subsets of TICs, respectively^3,14^. Overall, the results show that PISD regulates several *in vitro* phenotypes associated with EMT and TICs in breast cancer.

To investigate PISD in tumor formation, we injected 2 × 10^4^ SUM159-PISD-mCherry or control mCherry cells orthotopically into the mammary fat pads of female NSG mice. Cells expressed click beetle luciferase for bioluminescence imaging. Overexpression of PISD significantly reduced orthotopic tumor growth as quantified by imaging over 40 days (Fig. 4(d)). These data establish a novel function for PISD regulating tumor progression in breast cancer.

### PISD regulates mitochondrial morphology and function

**Figure 5:**
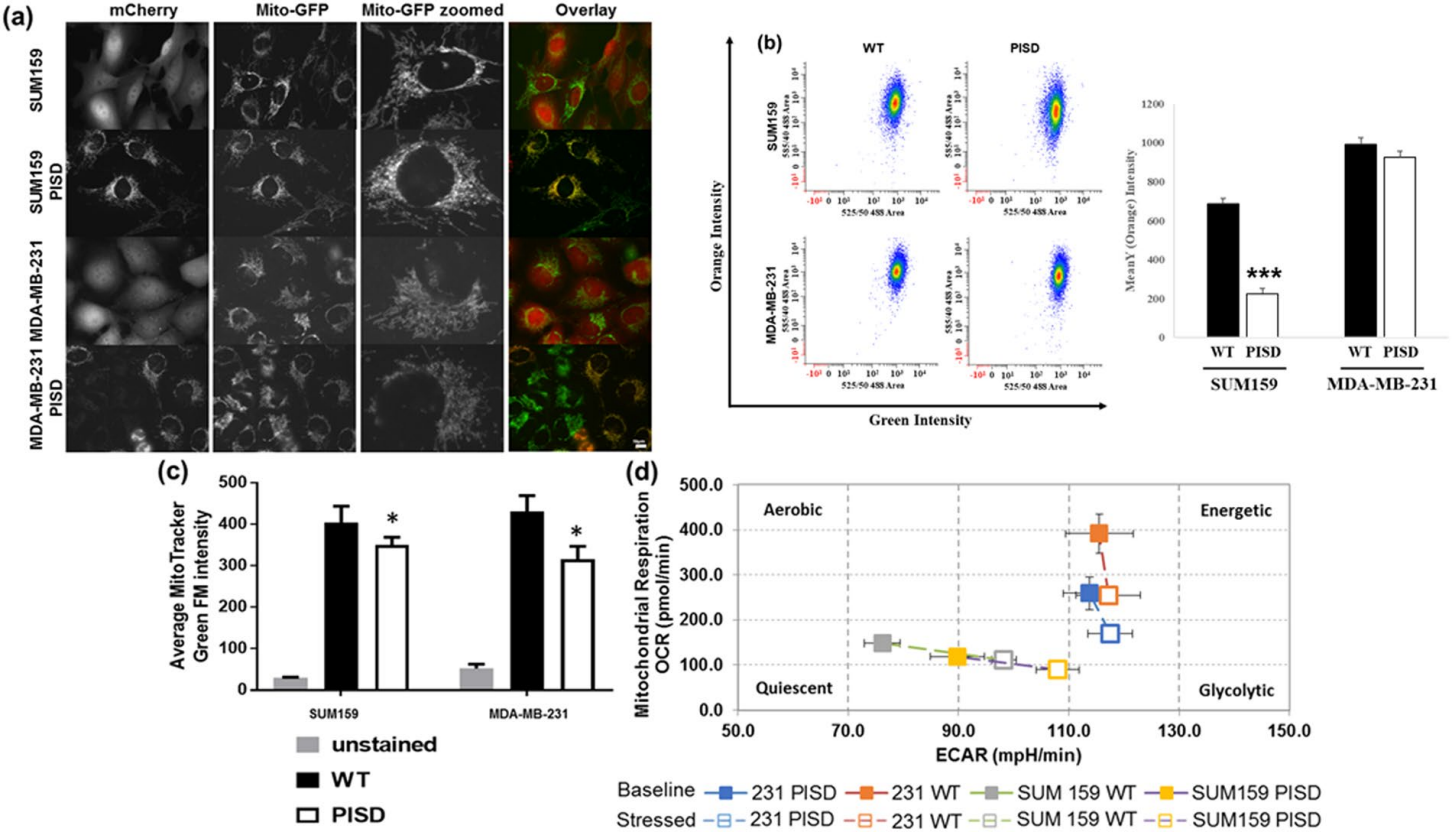
PISD overexpression drives cells away from TIC metabolism. (**a**) Fluorescence images of wild-type and stable PISD cells stably expressing mitochondrially-targeted GFP (Mito-GFP) show fragmentation of mitochondria in cells overexpression PISD. (**b**) Representative FACS analysis of SUM159 and MDA-MB-231 cells using the JC-1 metabolic dye. Quantification of the FACS plots is shown on the right. Graph shows mean orange intensity ± SEM. ***refers to P < 0.001. (**c**) FACS analysis of cells stained with Mito-Tracker Green shows reduced mitochondrial mass in cells overexpressing PISD. *refers to P < 0.05 (**d**) Representative metabolic flux assay shows reduced oxidative metabolism (OCR) in both cell lines and elevated glycolysis in SUM159 cells stably expressing PISD (n = 4).

Recent studies suggest that cancer stem cells may have increased numbers of mitochondria and are more reliant on oxidative metabolism than the bulk population^19,20^. As an enzyme in the inner mitochondrial membrane, we hypothesized that PISD regulates TICs through effects on mitochondrial morphology and metabolism. Fluorescence microscopy revealed expected localization of PISD-mCherry to mitochondria as defined by co-expression of mitochondrially-targeted GFP (Fig. 5(a)). We observed greater fragmentation of mitochondria in both SUM159 and MDA-MB-231 cells, suggesting that PISD regulates mitochondrial fission. Since mitochondrial fission typically decreases oxidative metabolism^21^, we investigated effects of several measures of mitochondrial function. SUM159-PISD-mCherry cells had significantly lower mitochondrial membrane potential than matched control cells under standard culture conditions, shown as a decrease in orange fluorescence (Fig. 5(b)). We observed a similar trend in MDA-MB-231-PISD-mCherry cells, although differences did not reach statistical significance. Expression of PISD also reduced overall mass of mitochondria (Fig. 5(c)). Finally, metabolic flux assays revealed decreased oxidative consumption rate (OCR) in both SUM159 and MDA-MB-231-PISD cells relative to control (Fig. 5(d)). SUM159-PISD cells also had a lower glycolysis as measured by extracellular acidification. Overall, these data establish that expression of PISD alters mitochondrial morphology and limits oxidative phosphorylation, providing a potential mechanism for inhibiting TICs.

## Discussion

Despite emerging evidence that TICs drive tumorigenesis and metastasis, current understanding of mechanisms regulating functions of these cells remains incomplete, hindering ongoing efforts to successfully target TICs therapeutically. An obstacle to progress in this field remains the challenge of isolating TICs directly by function rather than indirectly through marker-based approaches. To address this deficiency, we developed a microfluidic-based device to enrich TICs from bulk populations of cancer cells based on migration, a phenotype tightly linked to TICs through EMT. This device features several advances over standard microfluidic devices; (i) we scaled up to 900 migration channels per chip while maintaining uniform loading, markedly increasing throughput, (ii) we automated detection and localization of all cells in the device through a custom image analysis program, and (iii) we designed a highly reproducible cell retrieval scheme to isolate separate populations of migratory and non-migratory cells. Together, these novel advances enable the unique capability to isolate large numbers of migratory TICs for functional analyses and transcriptome sequencing. Our analyses showed significant enrichment of TICs in the migratory cell population, confirming the link between EMT and TICs in breast cancer. Furthermore, RNA sequencing data revealed that migratory TICs have a gene expression signature distinct from currently used markers in breast cancer (ALDH^br^ and CD24^−/low^/CD44^+^), allowing us to investigate new molecular regulators of TICs.

In this study, we identified PISD as a novel regulator of TICs. We found that PISD is significantly downregulated in migratory cells, and low expression correlates with reduced survival in patients with breast cancer. While enzymology of PISD in mitochondrial conversion of phosphatidylserine (PS) to phosphatidylethanolamine (PE) is well-characterized, few studies have associated PISD with cancer^22^, and no research has linked this gene with TICs. We found that transient or stable expression of PISD reduced the ability of cells to migrate, showing reversal of the EMT phenotype. Overexpression of PISD decreased the percentage of ALDH^br^ cells in both MDA-MB-231 and SUM159 cells. PISD also reduced the formation of tumor-spheres from single cells in only SUM159 cells. It should be noted that although MDA-MB-231 cells did not form mammospheres *in vitro*, these cells could form primary tumors, revealing a limitation of this assay. These data indicate that PISD reduces TICs. Concordant with these findings, PISD overexpression significantly reduced growth of orthotopic tumor xenografts in mice. These data are the first to show that PISD regulates TICs and tumor formation in breast cancer, highlighting the power of our migration-based functional assay to identify important regulators of TICs.

PISD localizes to the inner membrane of mitochondria, motivating us to investigate effects of stably overexpressing this protein on mitochondrial morphology and function. PISD-mCherry localized to mitochondria in cells stably expressing this fusion protein, resulting in increased mitochondrial fragmentation and decreased overall mass of mitochondria relative to vector control. These data are consistent with prior studies showing that bulk cancer cells have reduced mitochondrial mass relative to TICs^23–25^. SUM-159-PISD-mCherry cells also showed significantly reduced mitochondrial membrane. Differences in effects of PISD on mitochondrial membrane potential may reflect oncogenic mutations in both phosphatidyl-inositol-3-phosphate (PI3K) and Ras proteins in SUM-159 cells versus only a Ras mutation in MDA-MB-231 cells. Interestingly, knockdown or knockout of PISD in cells and mice, respectively, also produces similar changes in mitochondrial morphology and mass, while PISD^+/−^ mice were indistinguishable from wild-type^26^. These observations suggest that cells require a critical balance of PISD to regulate normal mitochondrial functions.

Consistent with previous studies^27^, we demonstrated that stable expression of PISD decreases oxidative phosphorylation (OXPHOS). Reprogramming of metabolism is essential to functions of TICs and cancer as a disease, and considerable evidence indicates that TICs preferentially utilize mitochondrial oxidative metabolism over glycolysis^28^. Moreover, prior research demonstrates that mitochondrial fusion promotes OXPHOS while fission drives glycolysis^29^, suggesting altered mitochondrial function in TICs. These observations are consistent with our data showing greater fragmentation of mitochondria with reduced OXPHOS, mitochondrial mass, and membrane potential in PISD cells. Therefore, PISD overexpression forces cells away from TIC metabolism, while a reduction in PISD as seen in the migratory cell population correlates with the metabolic state of TICs described previously^30^. Our data here show that PISD is a critical regulatory component of mitochondrial morphology and activity, providing a mechanism for effects of this protein on TICs.

In the current manuscript, we demonstrated the capability of our migration-based device to isolate TICs in triple negative breast cancer (TNBC), which encompasses molecular subtypes lacking expression of estrogen receptor (ER), progesterone receptor (PR), and amplification of Her2. EMT correlates particularly strongly with TICs in triple negative disease, the most aggressive subtype of breast cancer^31–33^. No effective molecularly targeted therapies exist for this subtype, unlike other subtypes of breast cancer. The close relationship between EMT and TICs in triple-negative breast cancer, combined with the compelling clinical need for better treatments, provides the driving rationale to focus our initial work on this subtype. Identifying key drivers of TICs in triple-negative breast cancer may open new options for molecularly-targeted therapy, meeting an obvious clinical need with clear benefits for improving patient care. Beyond triple negative breast cancer, cancer cells undergoing EMT acquire properties of TICs in several common epithelial malignancies, including prostate, lung, and ovarian cancer^34–37^. Therefore, our migration-based platform has the potential for broad applications as a functional assay for TICs across multiple cancers to better identify key regulators of TICs for mechanistic studies and drug development.

## Materials and Methods

### Microfluidic chip design and fabrication

The migration devices were fabricated from a single layer of PDMS (Polydimethlysiloxane, Sylgard 184, Dow Corning), which was fabricated on a silicon substrate by standard soft lithography, and a glass slide. Two masks were used to fabricate the multiple heights for main channels (40 µm height) and the migration channel (5 µm height). One device contains 900 migration channels (450 channels in one side), and the migration channel 30 µm in width, 5 µm height, and 1 mm in length. The PDMS layer was bonded to the glass slide after activated by oxygen plasma treatment (80 Watts, 60 seconds) to form a complete fluidic channel. The microfluidic chips were sanitized by UV radiation prior to use to ensure aseptic conditions. Before cell loading, collagen (Collagen Type 1, 354236, BD Biosciences) solution (1.45 mL Collagen, 0.1 mL acetic acid in 50 mL DI Water) was flowed through the device for one hour to coat collagen on the substrate to enhance cell adhesion. Devices were then rinsed with PBS (Gibco 10082) for one hour to remove the residual collagen solution.

### Cell Culture

We purchased MDA-MB-231 cells from the ATCC (Manassas, VA) and cultured cells in Dulbecco’s Modified Eagle Medium (DMEM) supplemented with 5% fetal bovine serum (FBS) and 1% Penicillin/Streptomycin (Pen/Strep) (Thermo Fisher Scientific, Waltham, MA). We obtained SUM159 cells from Dr. Stephen Ethier (now at The Medical University of South Carolina, Charleston, SC) and cultured cells in F-12 media supplemented with 10% fetal bovine serum, 1% Pen/Strep, 1% Glutamine, 5 μg/mL hydrocortisone, and 1 μg/mL insulin. We maintained all cells at 37 °C in a humidified incubator with 5% CO_2_.

### Cell Migration Assay and Cell Retrieval

Cells were harvested from culture plates with 0.05% Trypsin/EDTA (Gibco, 25200) and centrifuged at 1000 rpm for 5 min. Then, the cells were re-suspended in culture media to a concentration of 3 × 10^5^ cells/ml. 100 µL of this cell suspension was pipetted into upper and lower inlets. After 5 minutes, the cell solution in all inlets/outlets were replaced by 50 µL serum media. The device maintained static flow condition and stayed in incubator for 30 minutes to enhance cell adhesion, and we confirmed cell adhesion situation before doing the next step. 30 minutes are sufficient for the adhesion of MDA-MB-231 and SUM159 cell lines. Then, the cells were re-suspended in culture media to a concentration of 3 × 10^5^ cells/ml. 100 µL of this cell suspension was pipetted into the lower inlets. Then, the cell solution in the left inlet was replaced with 100 µL serum-free culture media, and 100 µL serum-free media with the indicated chemoattractant was applied to the central inlet to induce chemotactic migration. Due to the nature of diffusion, the concentration of the chemoattractant in the migration channel increases linearly along the channel from left/right channels to central channel. The detailed simulation and measurement were discussed in our previous work^15^. Then, the entire chip was put into a cell culture incubator. Migration distance was measured based on the final cell frontier (the cell migrating the farthest) of each migration channel after 24 hours of incubation without media replenishment. For data collection, cells were stained by LIVE/DEAD® Viability/Cytotoxicity Kit (Invitrogen, L3224) to distinguish live and dead cells. To have consistent results, we only use the data from central 300 (out of 450) migration channels. The difference in cell motility between upstream and downstream is less than 15% (Supplementary Fig. S6), and the results are consistent between devices (Supplementary Fig. S7). For separately retrieving highly-migratory and non-migratory cells from the device, we pipetted 100 µL of PBS to wash the microfluidic channels for 5 minutes, followed by 100 µL of trypsin for 5 minutes in incubator, and then applied mild negative pressure (~1,000 Pa) generated by a Pasteur pipette bulb to the outlets for 10 seconds. The devices were examined under microscope, and in our experiments, we found more than 95% of cells were retrieved from migration devices. Then, cells with the solution (~5 µL) in the outlets were collected, counted and re-suspended in 0.9% NaCl solution for mouse implantation or directly lysed for RNA purification for transcriptome sequencing.

### Transient transfection

We transiently transfected SUM159 cells or MDA-MB-231 cells (1.2 million) using Fugene-HD (Promega) with 10 µg of plasmid, either entirely TdTomato in pN1 (gift of A. Hoppe) or a mixture of 5 µg each of tdTomato plasmid and 5 µg of plasmid PISD PCMV6 (Origene RC200269) expressing human PISD. We sorted cells by flow cytometry for tdTomato fluorescence 48 hours after transfection and allowed the fluorescent cells to recover overnight in flasks before seeding migration assays on the next day, 72 hours after transfection.

### Lentiviral Vectors

We purchased an open reading frame for human PISD from Origene (RC200269) (Rockville, MD, USA) and amplified the cDNA with PCR primers 5′-ATGCGAATTCGCCACCATGTGTCAGTCAGAGGCGCGGCAAGG-3′ and 5′-TCTGCTCGAGCGGCCCACGCGTGAGCGAGCCCAGG-3′. We digested the PCR product with EcoRI and XhoI for ligation in-frame with a myc-tag and fluorescent protein mCherry amplified from plasmid pmCherry-C1 (Takara Bio USA, Mountain View, CA, USA) with primers 5′-GCCGCTCGAGCAGAAACTCATCTCAGAAGAGGATCTGGCAGTGAGCAAGGGCGAGGAGGATAA-3′ and 5′-GCATTCTAGACTACTTGTACAGCTCGTCCATG-3′. We digested the product with XhoI and XbaI for transfer into lentiviral expression vector pLVX-Puro (Takara Bio USA) under the control of a CMV promoter. We prepared a control lentiviral vector expressing mCherry only using PCR primers 5′ATGCTCTAGAGCCACCATGGTGAGCAAGGGCGAGGAGGATAAC-3′ and the same reverse primer as above. We cloned this PCR product with XbaI for cloning into the XbaI site of pLVX-Puro. We verified products by sequencing and expression of fluorescence from mCherry.

We produced recombinant lentiviruses and transduced target cells as described previously^38^. We first generated MDA-MB-231-CBG and SUM159-CBG cells stably expressing click beetle green luciferase as described previously and selected stable cells with blasticidin^39^. We subsequently transduced cells with either PISD-mCherry or mCherry viruses and sorted cells by flow cytometry for fluorescence of mCherry to obtain populations homogeneously expressing PISD-mCherry or mCherry only. The pLV-mitoGFP was a gift from Pantelis Tsoulfas (Addgene plasmid #44385), which expresses a Cox8 targeting sequence^40^. We sorted cells by flow cytometry for fluorescence of GFP to obtain homogeneous populations.

### Mouse Xenograft Implantation

The University of Michigan IACUC approved all animal procedures (protocol 00006795). The animals used in this study received humane care in compliance with the principles of laboratory animal care formulated by the National Society for Medical Research and Guide for the Care and Use of Laboratory animals prepared by the National Academy of Sciences and published by the National Institute of Health (Publication no NIH 85–23, revised 1996). We established orthotopic tumor xenografts in the fourth inguinal mammary fat pads of 9–17-week-old female NSG mice (Jackson Laboratory, Bar Harbor, ME, USA)^41^. For studies analyzing tumorigenesis of migratory versus non-migratory breast cancer cells, we recovered 100 migratory and non-migratory MDA-MB-231-CBG or SUM159-CBG cells from the migration device and implanted cells directly into mammary fat pads. To investigate effects of PISD overexpression on tumor growth, we implanted 2 × 10^4^ SUM159-CBG or SUM159-PISD-CBG cells orthotopically. We quantified tumor growth and metastasis by bioluminescence imaging as described previously^38^.

### Whole Transcriptome Next Generation Sequencing

We retrieved 1,000–2,000 migratory and non-migratory breast cancer cells (SUM159, MDA-MB-231) for next generation, whole transcriptome sequencing to identify differentially-expressed genes. The RNA was extracted using Norgen total RNA purification kit (Norgen, 17200), and the samples were processed by the University of Michigan Sequencing Core using a NEBNext® Ultra™ RNA Library Prep Kit. Each population is expected to have approximately 30 million (50 base pairs single-end) reads, and 3 bio-replicates were performed using 3 sequencing lanes. Reads were aligned using Bowtie and Tophat read aligners, and the transcriptome assembly and differential expression analysis were performed using Cufflink and Cuffdiff programs^42^. We identified genes with significant differences in expression between migratory and non-migratory cells as defined by p-value < 0.01 and fold change >2. For visualization of gene expression signature, R-based Seurat^43^ and Advaita iPathway were used in analysis.

### Prioritization of Differentially-expressed Genes

We first categorized genes significantly over- and under-expressed between migratory and non-migratory cells in both SUM159 and MDA-MB-231 breast cancer cells. Among overlapping genes (both over- and under-expressed in migratory cells from both cell lines), we used a publicly available database, kmplot.com^17^, to determine correlations with prognosis in breast cancer. To analyze the prognostic value of a particular gene, patient samples are split into two groups based on expression of that gene and compared by a Kaplan-Meier curve to calculate the hazard ratio with 95% confidence intervals and log rank P value. We selected genes for further evaluation based on concordance between changes in expression in migratory cells and worse survival in breast cancer (i.e. lower expression of a gene in migratory cells and correlation of low expression with significantly reduced survival in patients).

### Flow Cytometry

We quantified percentages of breast cancer TICs by activity of aldehyde dehydrogenase, using an ALDEFLUOR assay kit (Stemcell Technologies, Vancouver, Canada) per the manufacturer’s directions^9^. We used flow cytometry to sort for TICs or non-TICs based on aldefluor-positive or -negative populations, respectively. Alternatively, we sorted for TICs based on a CD24^low^/CD44^+^ phenotype (antibodies from Affymetrix eBioscience, San Diego, CA)^8^. We also utilized MitoTracker® Green FM (Invitrogen, M7514) and MitoProbe^TM^ JC-1 Assay Kit (Thermo Fisher, M34152) as per the manufacturer’s protocols to determine mitochondrial mass and membrane potential, respectively.

### Single Cell Sphere Formation Assay

To quantify the effect of PISD on single-cell-derived sphere formation, we loaded cells into a microfluidic chip with a single-cell suspension culture environment^18^. Before cell loading, we sanitized microfluidic devices using UV radiation and then primed devices with a 5% (w/w) PEO-terminated triblock polymer (Pluronic® F108, BASF) in DI water overnight before use. Channels were primed at least seven days after plasma activation for PDMS to restore its hydrophobicity and ensure quality F108 anti-fouling coating^44,45^. Before cell loading, we washed devices for one hour with phosphate-buffered saline (PBS). To prepare cells for experiments, we first stained SUM159 wild-type and PISD cells with green CellTracker dye (ThermoFisher, C2925) in suspension culture following the manufacturer’s protocol and then loaded cells into migration chips. During cell loading, we generated flow in the microfluidic device by a difference in liquid height (5 mm, 50 Pa) between the inlet and the outlet, capturing cells in each chamber within five minutes. After cell loading, we inverted the device to seed cells at the bottom of each well. We then cultured cells in custom serum-free medium for sphere culture. This serum-free medium contains MEBM (Lonza, CC-3151) supplemented with B27 (Gibco, 17504–044), 20 ng/mL bFGF (BD, 354060), 20 ng/mL EGF (BD, 354052), 5 μg/mL insulin (Sigma, I6634), 1 mM lipid concentrate (Gibco, 11905–031), 1 µg/mL hydrocortisone (Sigma, H4001), 7.8 µg/mL mercaptoethanol (Sigma, M3148), 3.9 µg/mL cholesterol (Sigma, C4951), and 1% penicillin/streptomycin (Gibco, 15070). We exchanged medium daily. We imaged chips immediately after loading (Day 0) and 14 days after loading (Day 14). We repeated staining with green CellTracker dye before imaging on Day 14 for sphere size analysis. We enumerated spheres with diameter larger than 40 µm were included for analysis. We previously have reported details of the device design and assay^18^.

### Metabolic Studies

We performed metabolic flux assays using a Seahorse Bioscience (Massachusetts, USA) XF^e^96 Extracellular Flux Analyzer and XF Phenotype test kit (Agilent, Santa Clara, CA) as described previously^46^. Briefly, we seeded 5 × 10^3^ cells per well one day before the assay, using injections of 0.25 µM FCCP and 1 µM oligomycin. We plotted data as the average of n = 24 wells over three measurement cycles and normalized to the total amount of protein in each well as measured by staining with sulforhodamine B (Luker, 1997 #1605).

### Image Acquisition

The microfluidic chips were imaged using an inverted microscope (Nikon). The bright-field and fluorescent images were taken with a 10x objectives and a charge-coupled device (CCD) camera (Coolsnap HQ2, Photometrics). A FITC/TRITC filter set was used for the fluorescent imaging of LIVE/DEAD® Viability/Cytotoxicity Kit (Invitrogen, L3224). Bright field imaging was performed using an exposure time shorter than 10 ms, and the fluorescent imaging was performed using an exposure time shorter than 100 ms, minimizing the phototoxic effect on cells. The microfluidic cell chamber array was scanned with a motorized stage (ProScan II, Prior Scientific). Before each scanning, the stage was leveled to ensure the image remained in the focus throughout the whole imaging area.

### Automated Cell Counting Program

The locations of live cells were obtained using a custom Matlab program developed by our lab^18^. For each LIVE/DEAD staining fluorescence microscopy image, there were three overlaid channels: bright field, FITC, and TRITC. This program obtained the fluorescent intensity value of each pixel on FITC channel and TRITC channel. Each pixel had a value ranged from 0 to 255 indicating its brightness, and the pixels with values greater than the pre-defined threshold were treated as “bright pixel” by the program. Any block containing over pre-defined number of “bright pixels” was counted as one valid cell, so that any noise, cell debris, and device defects were not included because of their small size or low fluorescent intensity. Dead cells were also excluded based on the signal in TRITC channel. In each migration channel, the program marked the live cells moving farthest as the migration frontier in that channel. The whole data analysis process takes less than 1 minute. We have validated the software by comparing manual distance measurement and computer-aided measurement. The difference between two approaches is under 3%

### Data analysis and processing

As cell migration results do not follow normal distribution, non-parametric Mann-Whitney U test were used for the comparisons of cell motility with a significance level of 0.05 considered statistically significant. *refers to P < 0.05, **refers to P < 0.01, and ***refers to P < 0.001. For sphere formation and other experiments, two-tailed, unpaired student’s t-tests were used. The data in the bar graphs (created using Excel) are presented as mean ± SD. The Box graphs were plotted using Origin 9.0. The bottom and top of the box are the first and third quartiles, and the band inside the box is always the second quartile (the median). The ends of the whiskers represent the 5th percentile and the 95th percentile. The square inside the box indicates the mean, and the x outside the box indicates the minimum and maximum of all the data.

## Electronic supplementary material

Supplementary Information

Supplementary Video 1

Supplementary Video 2

Supplementary Video 3

Supplementary Dataset 1
