# Supplementary material for: Functional Isolation of Tumor-Initiating Cells using Microfluidic-Based Migration Identifies Phosphatidylserine Decarboxylase as a Key Regulator
Source: Sci Rep. 2018 Jan 10;8:244. doi: 10.1038/s41598-017-18610-5 (PMC5762897; doi:10.1038/s41598-017-18610-5)
Supplement: Supplementary file 1 — Supplementary Information [file 41598_2017_18610_MOESM1_ESM.pdf]

Title: Functional Isolation of Tumor-Initiating Cells using Microfluidic-Based Migration Identifies Phosphatidylserine Decarboxylase as a Key Regulator

Authors and affiliations:

Yu-Chih Chen*[1, 2, 3], Brock Humphries[4], Riley Brien[1], Anne E. Gibbons[4], Yu-Ting Chen[5], Tonela Qyli[4], Henry R. Haley[4], Matthew E. Pirone[4], Benjamin Chiang[4], Annie Xiao[4], Yu-Heng Cheng[1], Yi Luan[1], Zhixiong Zhang[1], Jason Cong[5], Kathryn E. Luker[4], Gary D. Luker[*4,6,7], and Euisik Yoon[*1, 7]

[1] Department of Electrical Engineering and Computer Science, University of Michigan, 1301 Beal Avenue, Ann Arbor, MI 48109-2122;

[2] Comprehensive Cancer Center, University of Michigan, 1500 E. Medical Center Drive, Ann Arbor, MI 48109, USA;

[3] Forbes Institute for Cancer Discovery, University of Michigan, 2800 Plymouth Rd., Ann Arbor, MI 48109, USA;

[4] Center for Molecular Imaging, Department of Radiology, University of Michigan, 109 Zina Pitcher Place, Ann Arbor, MI 48109-2200, USA;

[5] Computer Science Department UCLA, Boelter Hall, Los Angeles, CA 90095-1596

[6] Department of Microbiology and Immunology, University of Michigan, 109 Zina Pitcher Place, Ann Arbor, MI 48109-2200, USA;

[7] Department of Biomedical Engineering, University of Michigan, 2200 Bonisteel, Blvd. Ann Arbor, MI 48109-2099, USA

*Corresponding authors

Yu-Chih Chen
1301 Beal Avenue, Ann Arbor, MI 48109-2122, USA
Tel: 734-272-7113; E-mail: yuchchen@umich.edu.

Gary D. Luker
University of Michigan Medical School
109 Zina Pitcher Place
A526 BSRB
Ann Arbor, MI 48109-2200, USA
Tel: 734-763-5476; E-mail: gluker@umich.edu

Euisik Yoon
1301 Beal Avenue, Ann Arbor, MI 48109-2122, USA
Tel: 734-615-4469; E-mail: esyoon@umich.edu.

Keywords:

Cell Migration; Tumor-initiating Cells; PISD; Microfluidics; Whole Transcriptome Sequencing; Breast Cancer.

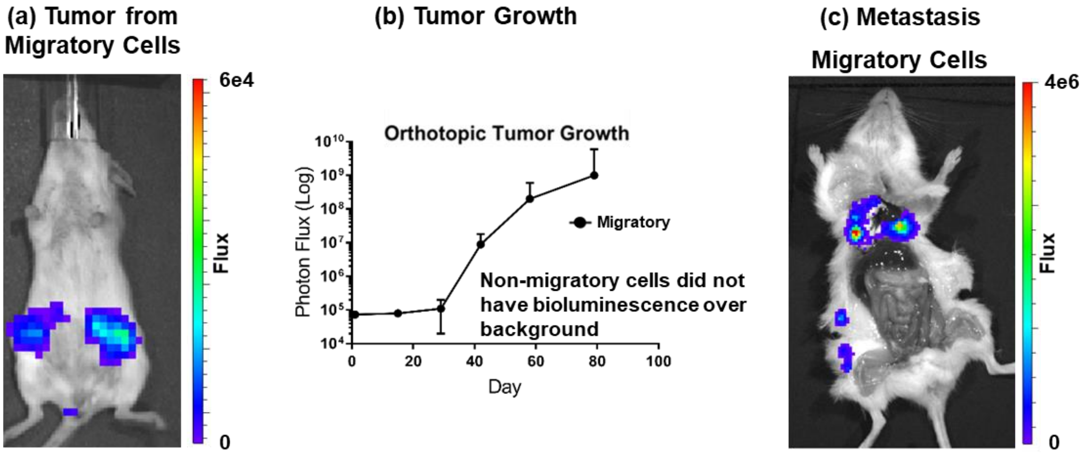

Supplemental Fig. S1. Increased tumor initiation, growth, and metastasis of SUM159 migratory cells. (a) Only the migratory SUM159 breast cancer cell produced tumors. Representative bioluminescence image of a female NSG mouse 85 days after orthotopic implantation of 100 migratory SUM159 cells recovered from the migration device. Scale bar denotes range of photons displayed on pseudocolor scale with red and blue denoting highest and lowest values, respectively. Mouse was injected with luciferin diluted 1:100 in PBS. (b) Tumor growth of the highly-migratory SUM159 cells as quantified by bioluminescence. Graph displays mean values ± SEM for bioluminescence from migratory cells. Only migratory cells produced bioluminescence above background. (c) Representative bioluminescence image showing metastasis of a mouse injected with 100 SUM159 migratory cells. Only the migratory SUM159 cells metastasized.

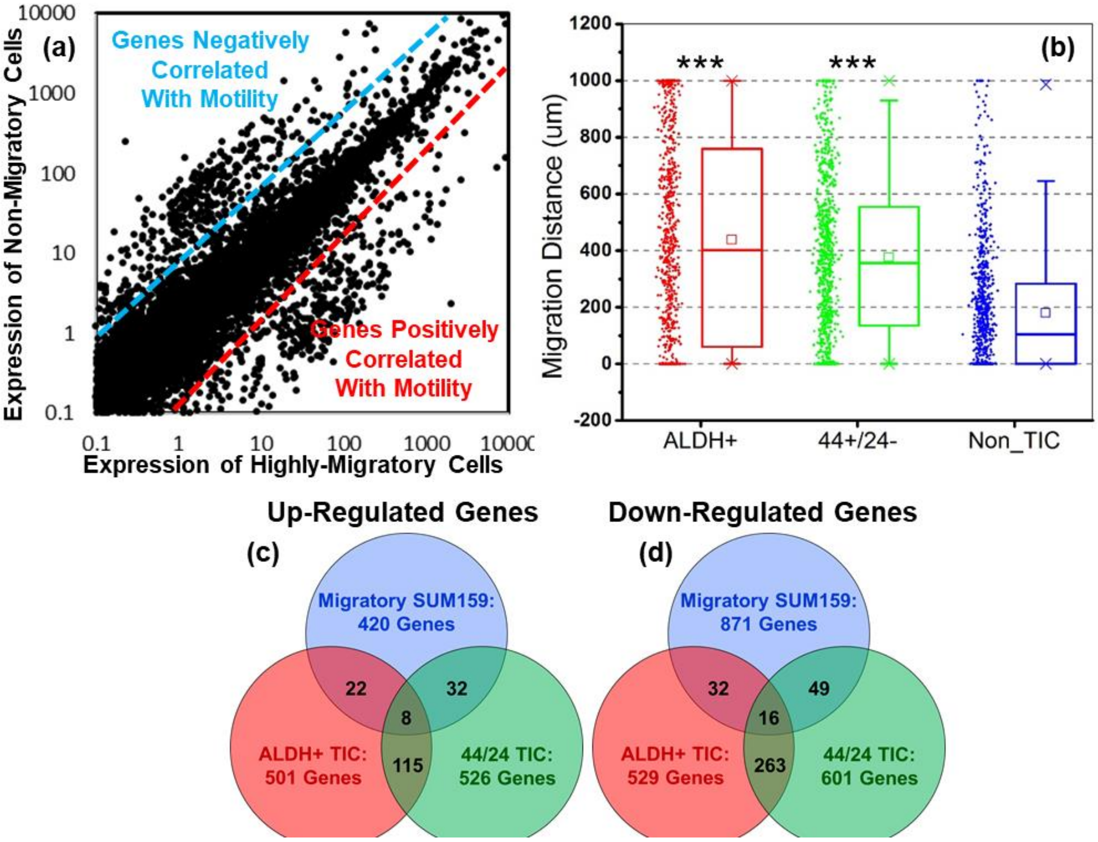

Supplemental Fig. S2. Whole transcriptome sequencing reveals genes differentially expressed between SUM159 migratory and non-migratory cells. (a) Gene expression of migratory and non-migratory MDA-MB-231 cells determined by next generation sequencing. Each dot represents a gene. Genes in the upper left and lower right corners correlate negatively and positively with migratory cells, respectively. (b) Heat map shows expression of top 100 elevated and suppressed genes of migratory and non-migratory SUM159 cells in three replicates (n=3). Each column is a sample, and each row is a gene. Red color represents high expression, and blue color represents low expression. (c) 5% serum chemo-attraction migration assay of $ALDH^{+}$, $CD24^{low}/CD44^{+}$ and non-TIC of SUM159 cells. Both $ALDH^{+}$ and $CD24^{low}/CD44^{+}$ cells have higher motility than non-TICs. (n=1,200 channels). *** refers to $P < 0.001$. (d-e) Comparison between significantly up-regulated and down-regulated genes of $ALDH^{br}$ and $CD24^{-/low}/CD44^{+}$ cells and the presented TICs identified by cell migration.

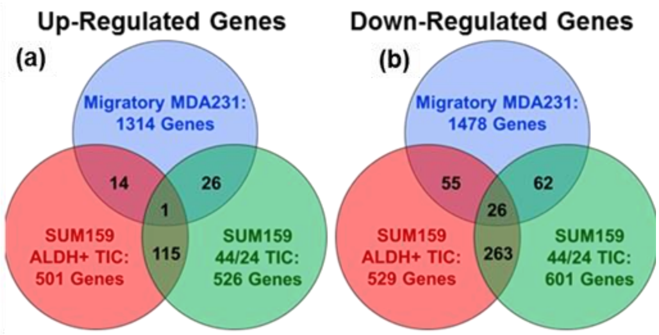

Supplemental Figure S3. Comparison between significantly up-regulated and down-regulated genes of SUM159 ALDH$^+$ and CD24^{low}/CD44$^+$ cells and the presented MDA-MB-231 TICs identified by cell migration. Genes identified for SUM159 ALDH^{br} and CD24$^{-/low}$/CD44$^+$ were previously identified[16].

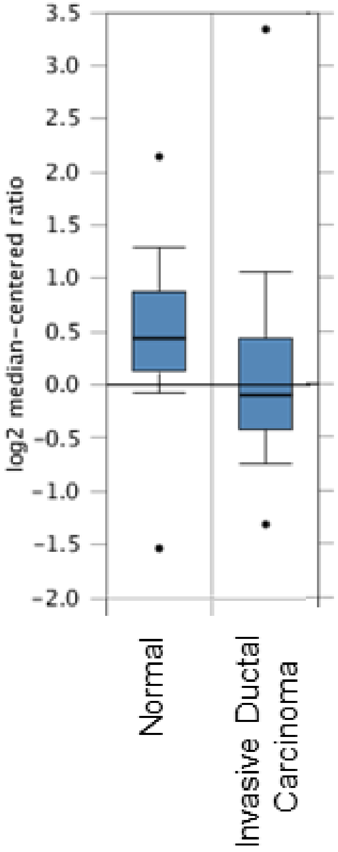

Supplemental Fig. S4. PISD is lower in invasive ductal carcinoma than normal breast tissue. Adapted Oncomine data show a wide range of expression of PISD in invasive breast cancer relative to normal breast tissue. The expression values are taken from the TGCA mRNA microarray. Normal: n=61, Invasive Ductal Carcinoma: n=389.

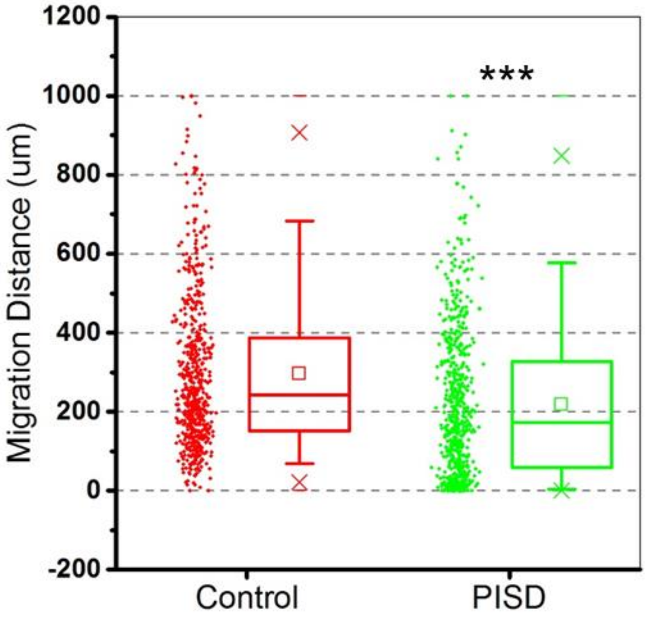

Supplemental Fig. S5. Transient transfection of PISD reduces migration of SUM159. (b) We transfected SUM159 cells with tdTomato (control) or an under-expressed gene in migratory cells (PISD). Transient overexpression of PISD significantly reduced migration of SUM159 cells toward 5% serum. (n = 1,200 channels). *** refers to $P < 0.001$. We verified expression by RT-qPCR (not shown).

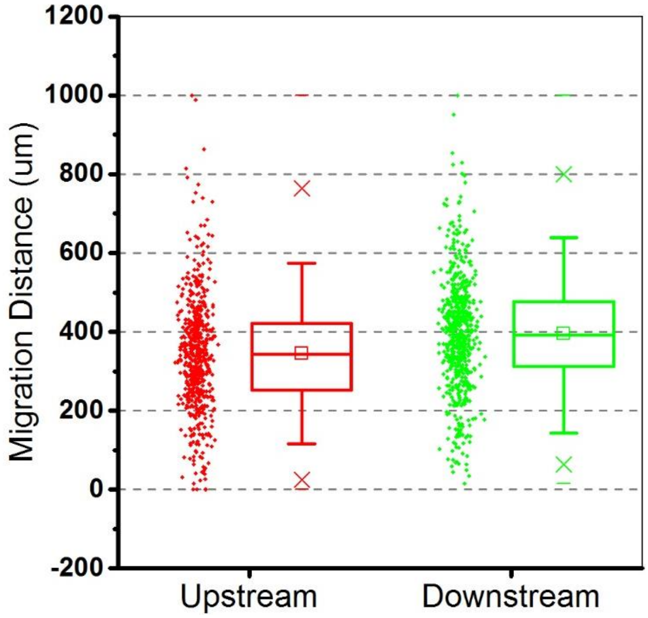

Supplemental Fig. S6. The difference in cell motility between upstream and downstream of the migration device is less than 15%. SUM159 cells were attracted toward 5% serum. (n = 600 channels per condition from 4 replicates).

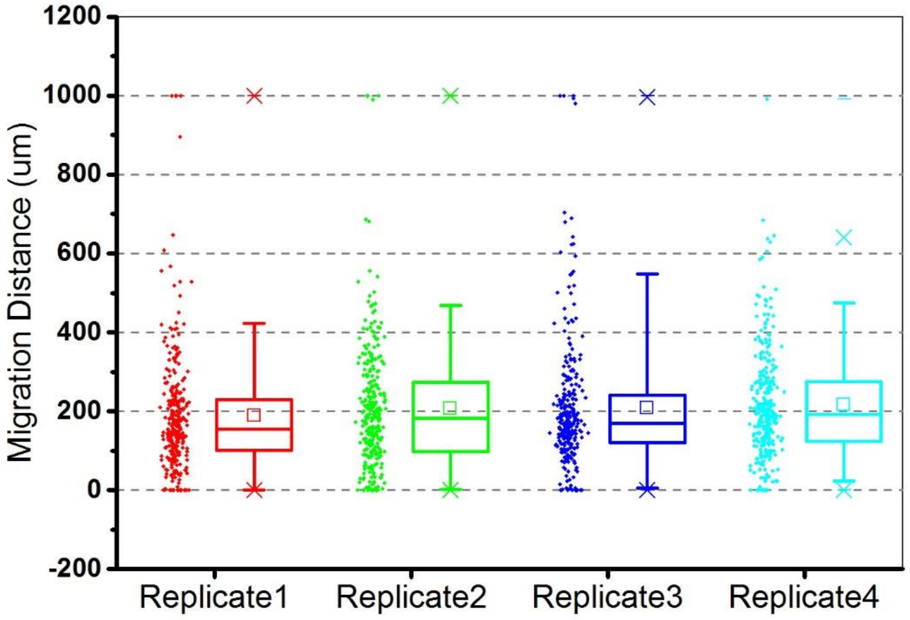

Supplemental Fig. S7. Low variation between migration experiment replicates. SUM159 cells were attracted toward 5% serum. (n = 300 channels per replicate).
